# Sit and face the world: ontogenetic adaptation in infant vocal production and visual attention during the transition to independent sitting

**DOI:** 10.1186/s40359-025-02645-9

**Published:** 2025-04-01

**Authors:** Zuzanna Laudańska, Anna Malinowska-Korczak, Karolina Babis, Szymon Mąka, Itziar Lozano, Peter B. Marschik, Dajie Zhang, Katerina Patsis, Magdalena Szmytke, Monika Podstolak, Weronika Araszkiewicz, Przemysław Tomalski

**Affiliations:** 1 Institute of Psychology, Polish Academy of Sciences, Warsaw, Poland; 2 Department of Child and Adolescent Psychiatry, University Hospital Heidelberg, Heidelberg University, German Center for Mental Health (DZPG), Heidelberg, Germany; 3 iDN – interdisciplinary Developmental Neuroscience, Division of Phoniatrics, Medical University of Graz, Graz, Austria; 4 Center of Neurodevelopmental Disorders (KIND), Department of Women’s and Children’s Health, Centre for Psychiatry Research, Karolinska Institutet & Region Stockholm, Stockholm, Sweden; 5 Child and Adolescent Psychiatry and Psychotherapy, University Medical Center, German Center for Child and Adolescent Health (DZKJ) and Leibniz ScienceCampus Primate Cognition, Göttingen, Göttingen, Germany; 6 Institute of Psychology, Faculty of Philosophy and Social Sciences, Nicolaus Copernicus University in Toruń, Toruń, Poland

**Keywords:** Infants, Eye-tracking, Head-mounted eye-tracking, Wearables, Inertial motion units, Motor development, Attention to faces, Vocalizations, Parent-child interactions

## Abstract

**Background:**

Motor milestones are not only indicators of developmental progress, but they also open up new opportunities for infants to interact with the environment and social partners, as the development of motor, social, and language skills is tightly interconnected in infancy. This study will investigate how the transition to independent sitting relates to key areas of socio-communicative development in infancy: vocal production and visual attention.

**Methods:**

This study addresses the relationship between sitting acquisition and social cognition skills in infancy. It will allow for comparing if infant motor development, vocalizations, and visual attention undergo developmental changes in parallel or whether they have intertwined trajectories. We will conduct a longitudinal study using a milestone-based approach to account for individual differences in relation to the timing of motor milestone acquisition. We will invite parent-infant dyads to the lab when infants are at different stages of independent sitting acquisition: non-sitting, attempting-sitting and expert-sitting. Infants’ attention toward faces and toys will be measured with a wearable eye-tracker during free-flowing dyadic interactions with their caregivers. During the same interactions, infant vocalizations will also be recorded and analyzed. Additionally, screen-based eye-tracking will be used to precisely assess changes in infants’ attention to the mouth area of the speaker.

**Discussion:**

Altogether, this study will provide a unique dataset that tracks the cross-dependence of motor, visual and vocal developmental trajectories. It will have the potential to inform future studies of neurodevelopmental disorders such as autism that are characterized by socio-communicative challenges.

## Introduction

Neurodevelopmental conditions such as autism spectrum disorder (ASD) are often characterized by socio-communicative challenges (e.g [1–3]). One of the early differences in the context of socio-cognitive and communicative development is visual attention to faces. Chawarska et al. [4] and Jones et al. [5] found that six-month-old infants at elevated likelihood for ASD (with an older sibling with autism, EL-infants) – who eventually developed the condition themselves – tended to look less at the face of a speaker than those who did not, and compared to low-likelihood infants (LL-infants). Shic et al. [6] replicated this finding, but again only for speaking faces, not silent ones. Results from a study on infant-parent interactions showed that, regardless of later diagnosis, EL-infants, at a group level, looked less often at their parents’ faces than LL-infants [7]. In contrast, Elsabbagh et al. [8] found *increased* visual attention to faces presented on a screen in EL-infants as a group, irrespective of their later autism diagnosis, compared to LL-infants. Furthermore, a live eye-tracking study found that infants with and without a subsequent autism diagnosis differ very little in how much they look toward faces overall [9]. These puzzling differences indicate that the developmental variability in visual attention to faces – especially in naturalistic social interactions – is not yet fully understood, either in autism or in typically developing infants [10]. Moreover, some results from screen-based experiments, which limits ecological validity, may not reflect infants’ visual attention during real-life social interactions (see discussion in [11]), Importantly, visual attention to faces can also be linked to changes in gross motor development.

### Acquisition of sitting skills in relation to visual attention and vocal production

One of the key gross motor achievements changing the visual perspective of young infants is the developmental ability to sit independently [12, 13]. Infants usually start sitting independently between 5 and 8 months of age, with large disparities in the time of its onset (WHO Motor Development Study [14]). While sitting, infants are more likely to have toys in their view [12, 15], and they are also more in the center of their field of view [12, 16]. Similarly, sitting infants are more likely to see a caregiver’s face [15, 17], and the episodes of parent-infant mutual gaze and joint attention also happen more often when infants sit [17]. The caregivers are more likely to provide opportunities for learning when infants are sitting independently than when they are in other positions [13]. Interestingly, there are differences in how caregivers play with infants depending on their sitting proficiency. Supported sitting provides infants less of a boost to cognitive opportunities than independent sitting, potentially due to the fact that when caregivers provide postural support, they are likely to be positioned behind their infants, decreasing chances for face-to-face engagement and joint attention [13]. Accessibility of important social information from caregivers’ faces is easier during face-to-face play than during supported sitting [18].

Sitting has since long been postulated to relate to advances in vocal development. Yingling [19] documented significant associations between the onset of unsupported sitting and advances in consonant-vowel vocalizations in the first year of life, which are important precursors to speaking first words. Gallen et al. [20] reported that more advanced gross motor skills are linked to more advanced prelinguistic development and social development. Similarly, positive associations between gross motor and communication development were found in very preterm infants, with higher expressive language scores and more vocal production characterizing infants mastering unsupported sitting [21]. Furthermore, the earlier-than-average development of sitting skills (from 3 to 5 months of age) was predictive of subsequent higher receptive language scores at later time points (at 10 and 14 months of age), even while controlling for overall gross motor development [22]. However, it is unknown whether the acquisition of independent sitting skills is related to advances in infants’ vocal production.

### Acquisition of sitting skills in relation to visual attention to mouth

Screen-based eye-tracking research showed that the way in which infants focus on different facial areas (eyes, mouth) seems to undergo significant changes around the age when they start sitting independently. Infants’ visual attention to the articulating mouth increases between 4 and 8 months of age [23–27] as it provides audio-visual speech cues, which are highly relevant for acquiring phonetic, phonological, and vocabulary skills [27–30]. It has also been hypothesized that this increase in visual attention to the mouth may also be related to vocal development [23, 31], as infants use visual speech cues of talking faces as feedback for their own speech production.

### Consequences of delays in motor development for socio-communicative skills

Due to these important cascading effects on socio-communicative skills, delayed acquisition of sitting can have then crucial consequences for overall child development. Delayed acquisition of independent sitting might be an indicator of atypical development, but it is a sign of disintegrity of the developing system and not a condition-specific marker. For example, children with cerebral palsy (e.g [32]). Down syndrome (e.g [33]), and Rett syndrome (e.g [34, 35]), as well as infants who later receive an ASD diagnosis (e.g [36, 37]), might exhibit delays in sitting acquisition. Even though the delayed sitting acquisition is not the earliest marker of motor atypicality (earlier ones include, for example, differences in general movement patterns, maintaining stable head position, delayed acquisition of rolling from prone to supine and supine to prone, e.g [38–40], it is one of the red flags that parents can easily identify themselves and seek professional assessment and support. It is especially important in the case of neurodevelopmental conditions that usually get diagnosed later in toddlerhood or early childhood but could hugely benefit from early identification and intervention or professional support, such as ASD [41]. Even though postural deficits are not a primary diagnostic criterion for ASD, evidence suggests that they are part of the overall prodromal ASD profile (see [42–45] for reviews). Concerns related to the motor domain are also reported by parents of EL-infants (e.g [46]).

### Tracking developmental transitions of multiple skills

As each new posture is initially unstable (as measured behaviorally or kinematically) and requires practice (involving multiple failed attempts), it is important to track changes across the process of learning a new motor skill. For example, expert-sitters can adapt their postural control to maintain balance on both compliant and firm surfaces [47], but similar variability can be difficult for infants who just started to sit independently (new-sitters). For them, the challenge of staying upright takes a lot of attentional resources, minimizing their engagement in other behaviors, such as reaching for objects [48, 49]. New-sitters, during their first attempts, need to learn how to maintain their balance to avoid falling and adjust the coordination of the upper limbs with the demands of sitting upright (e.g [37]).,. Once the novel skill is mastered, it requires less attentional resources and allows for the simultaneous performance of other actions [50]. Thus, behavioral flexibility increases with sitting experience [51].

In this study, we align the testing schedule with the onset of independent, arms-free sitting rather than chronological age in order to explore how visual attention and vocal production change across the process of learning a new motor skill. We will control for chronological age, but the milestone-based approach aims to account for individual differences in the timing of sitting acquisition. Recently, Gallen et al. [20] showed that age-adjusted relative advances in motor development are linked to concurrent prelinguistic and social development, supporting the idea of developmental interaction across neurocognitive domains. In this study, we aim to capture such developmental interactions across neurocognitive domains but center them around a specific motor milestone to better understand the age-driven vs. posture-driven effects.

### The current study

The acquisition of independent sitting transforms infant visual attention to faces [12, 15] in general but it is not clear whether it also contributes to the increase in visual attention to the mouth area as a source of audiovisual speech cues. The second related but unexplored issue concerns how the acquisition of sitting may alter the allocation of attention to speaking faces relative to other attractive visual objects during free-flowing social interactions. The proposed study, for the first time, combines the measurement of visual attention in a screen-based task with the measurement of attention during free-flowing social interaction in a design that tests their dependence on sitting acquisition. The acquisition of sitting potentially reorganizes multiple aspects of speech and face processing [52].

We aim to examine developmental trajectories in a cohort of infants with a low likelihood of ASD first to determine whether changes in vocal production and visual attention to faces occur simultaneously in relation to the acquisition of independent sitting. Understanding these trajectories first in typically developing infants will allow us to establish a baseline prior to any studies with EL-infants.

Pilot data collection and finalization of the study design were conducted between 13.07.2023 and 09.02.2024. The recruitment and data collection are ongoing. Data collection started on 16.02.2024. The data analysis has not started. The expected completion date of the recruitment is the end of August 2025.

### Study objectives

#### Objective 1

To investigate how the transition to independent sitting changes infants’ visual attention to faces and toys during free play.

Prediction: Due to fewer postural constraints and less effort to maintain balance, infants, after learning to sit independently, would look longer at the caregiver’s face and toys than before acquiring this motor milestone [15, 17]. However, during the phase transition (attempting-sitting), infants’ look less toward faces and toys would be shorter than before and after the acquisition of sitting due to difficulties in maintaining a stable body position.

#### Objective 2

To investigate how the transition to independent sitting influences infants’ visual attention to mouth in a screen-based eye-tracking task.

Prediction: Increasing experience with viewing faces in an upright orientation, infants’ visual attention to the mouth while watching talking faces in a free-viewing task gradually increases as they gain sitting proficiency.

#### Objective 3

To investigate how the transition to independent sitting associates with infants’ vocal production.

Prediction: After learning how to sit independently, infants vocalize more and produce more advanced vocalizations (such as canonical babbling) than before acquiring this motor milestone [53, 54]. However, during the transitional phase (attempting-sitting), infants vocalize less due to difficulty in allocating attention to multiple tasks during the acquisition of a novel skill [50].

### Methods and analysis

#### Sampling plan

Participants are recruited through snowballing, using ads on social media (Facebook, Instagram) and parenting blogs in Poland. The participants are recruited from the metropolitan area of Warsaw (a city with over 1.8 mln inhabitants). We expect the majority of participants to come from families with a middle-class background and mean-to-high socio-economic status – we will collect SES-related information during the first visit. For their participation, infants receive a diploma and a small gift (a baby book). Infants’ caregivers provide written informed consent before data collection. This study has been approved by the Research Ethics Committee at the Institute of Psychology, Polish Academy of Sciences.

### Sample size

GPower analysis indicates that based on the standard repeated-measures design for a moderate effect size, a minimum sufficient number of participants should be ≈ 30. We aim to recruit 60 infants to overcome the attrition rate and missing data, which are common issues in infant studies (e.g [55]).

### Study design

Our project follows a milestones-based longitudinal experimental design in which infants are invited to the lab around the time they approach the transition to independent sitting. We use a milestone-based approach (e.g [37])., rather than an age-range approach to account for large individual differences in the timing of motor milestone acquisition. Parents fill in weekly surveys about the gross motor development of their infants (illustrated item list for pre-screening if anything has changed in the past week), and these answers are used to schedule lab visits. Furthermore, during each lab visit, infants’ stage of sitting acquisition will be confirmed using two approaches. First, we will use the standardized assessment, following the Alberta Infant Motor Scale [56] to measure the infants’ *capacity* to sit alone. Second, we will measure their *performance*, so the actual time spent by infants in a sitting position during infant-parent interactions by applying the posture classification algorithm based on IMU data [57], recorded during lab visits.

Furthermore, infants’ ages will be added to the analyses to control for the interaction between age-related and motor-milestone-related effects.

Each infant will participate in 3 or 4 visits (depending on the individual development with respect to independent sitting):

- pre-sitting (1 visit): when an infant stays comfortably in prone while one hand is free for manipulating objects.
- attempting-sitting: transition to independent sitting (1 or 2 visits, depending on the progress of the acquisition of sitting) when an infant is able to sit with support while maintaining the head in the midline; an infant can also attempt the position of brief independent sitting without arm support but cannot be left alone in sitting position (first unstable attempts of independent sitting).
- expert-sitting (1 visit): an infant can comfortably sit independently without any support, can be left alone in sitting, and play with toys in this position.

The above-mentioned definitions are based on the Alberta Infant Motor Scale [56].

### General procedure

During each lab visit, interactions will be recorded in an infant-friendly laboratory room on a carpeted play area. Upon the family’s arrival, an experimenter will explain the study protocol and obtain informed parental consent. Once the infant is familiarized with the laboratory, a screen-based eye-tracking task will start (see detailed procedure below). After the screen-based eye-tracking task, the wearable motion trackers will be put on the infant (on arms and legs) and caregiver (on arms), and a wearable eye-tracker will be positioned on the infant’s head. Then, a set of parent-child interaction tasks with different sets of age-appropriate toys will take place. The caregivers will not receive any instructions other than to play with their children as they typically would do at home. To investigate the cross-situational stability of effects, there will be 4 different interactive tasks during each meeting: book-sharing, rattle-shaking, playing with manipulative toys, and playing with toys that are aimed at eliciting parental action demonstration, each lasting around 5 min (see detailed description below). The order of plays will be randomized between participants and testing sessions. Then, there will be a free-play task without wearable motion trackers and wearable eye-tracker. All tasks will be recorded using 3 remote-controlled HD CCTV cameras (Axis), and the sound recording synchronized with the video for vocal production analysis will be carried out with a high-grade cardioid membrane condenser microphone (Sennheiser e914) placed underneath one of the cameras. Finally, a standardized assessment of gross motor development will be conducted, following the Alberta Infant Motor Scale [56]. Additionally, the caregivers will fill out several questionnaires and answer questions related to demography and socioeconomic status (see **Control Variables** section for an overview).

### Interactive tasks

- **Rattle-shaking**: duration of approximately 5 min; the infant and the parent were given two maracas rattles and two small and light barbell rattles.
- **Book-sharing**: duration of approximately 5 min; the infant and the parent were given two small baby books and one small book with tactile elements.
- **Playing with small manipulative toys (infant object exploration)**: duration of approximately 5 min; the infant and the parent were given a sensory pop-it toy, a sensory toy with propellers that spin down a corkscrew pole, a wooden wiggly worm and a sensory bee toy with different silicone elements.
- **Playing with larger manipulative toys (parental action demonstration)**: duration of approximately 5 min; the infant and the parent were given a set of toys that provided multimodal feedback (sounds, movements) and were designed for older children, so infants needed parental action demonstration: a silicone-pulling toy with textured cords, a spinning toy with small balls inside, a sensory-exploration toy with elements with different textures that can be pushed, spun or clicked and make different sounds, a roll-along pull toy telephone with a smiling face that makes chattering sound and the eyes move up and down when pulled.
- **Free Play (without wearables)**: duration of approximately 5 min; the infant and the parent were given toys encouraging open play: a set of stacking cups, a wooden box with a drawer and a wooden ball, a rattle, a set of 4 plastic flowers that swing freely on the lightweight ring, a small finger puppet book, and a small cloth book with crinkle fabric and animal tails with different textures.

### Objective 1. To investigate how the transition to independent sitting changes infants’ visual attention to faces and toys during free play

#### Mobile eye-tracking procedure

**Fig. 1:**
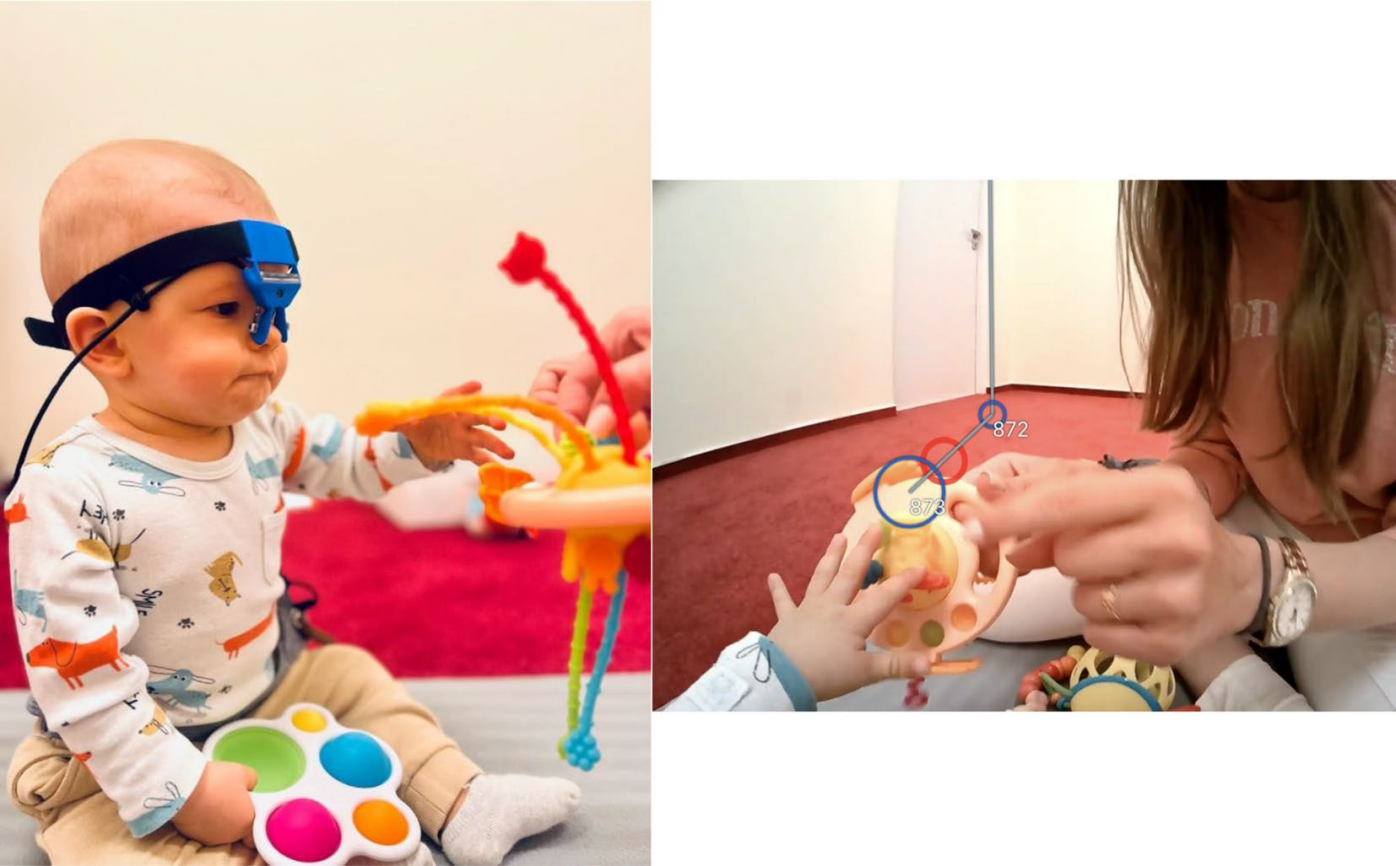
Positioning of the wearable eye-tracker on an infant’s head (left) and an example frame from the eye-tracking recording (right). The images are displayed with permission from the caregiver

The experimenter will put the wearable eye-tracker Pupil Labs Neon with a “Ready Set Go frame” on the infant’s head (**see** Fig. 1). The binocular eye-tracking is based on real-time neural network technology. The eye-tracking process relies on two infrared eye cameras recording at 200 Hz and one scene camera recording at 30 Hz and does not require calibration. The device also includes an Inertial Motion Unit with an accelerometer, magnetometer, and gyroscope. Initial pilot data collection showed great tolerability by infants. The experimenters will position the eye-tracker on the infant’s head so that the eye cameras are pointing toward the eyes and the scene camera is directly above the eyes and centered. Once the eye-tracking equipment is properly set up, the dyads will participate in 4 types of play activities (see **General procedure** section). After the session, the recordings will be saved for further analysis.

#### Mobile eye-tracking data analysis - visual attention to faces

To assess developmental changes in infants’ visual attention to faces during play with caregivers, we will automatically analyze the data from the mobile eye-tracking and the infant’s first-person video of the scene. In the first step, the % of recording duration when the caregiver’s face is visible in the infant scene will be examined using an artificial intelligence (AI) face detector. We plan to use RetinaFace [58], but since research in this area is developing at a very high pace, we will review the available options regularly.

We will map gaze data onto faces that appear in the visual scene. Then, we will analyze the percentage of recording duration where fixations on faces occur. Moreover, we will extract the percentage of time the infant fixated on faces out of the time when the faces were detected in the infant scene camera (so if the face is visible in the field of view, whether the infant looks at it).

#### Mobile eye-tracking data analysis - visual attention to toys

To assess developmental changes in infants’ visual attention to toys during play with caregivers, the infant’s first-person video of the scene with overlaid fixation data will be downloaded for further annotations. Infants’ visual attention will be then manually annotated using ELAN software (https://archive.mpi.nl/tla/elan). Trained coders will label the segments based on the crosshair’s location, indicating whether the infant looked at any of the toys (each toy will be considered a separate Area-of-Interest (AOI). Coders will also annotate moments when the object of fixation cannot be readily discerned. Furthermore, coders will also exclude moments when the eye-tracker was being adjusted after being bumped or moved by the infant.

### Objective 2. To investigate how the transition to independent sitting influences infants’ visual attention to mouth in a screen-based eye-tracking task

#### Screen-based eye-tracking procedure

The eye-tracking task is the first task during each visit (after the period of getting familiar with the testing room). During the eye-tracking task, infants will sit on their caregiver’s lap approximately 60 cm from the monitor presenting stimuli. The eye-tracking data will be collected on a Gazepoint GP3 eye-tracker recording at 60 Hz. The stimuli will be presented on the 23.8” monitor (1920 × 1080 pixels, 75 Hz refresh rate) using PsychoPy software (version 2021.1.4 [59], running on a laptop with Windows 11 operating system. The standard 5-point calibration procedure will be conducted at the beginning of each eye-tracking session using Gazepoint software (as recommended by the manufacturer). We will use the eye-tracking data if 4 or 5 points were successfully calibrated.

**Fig. 2:**
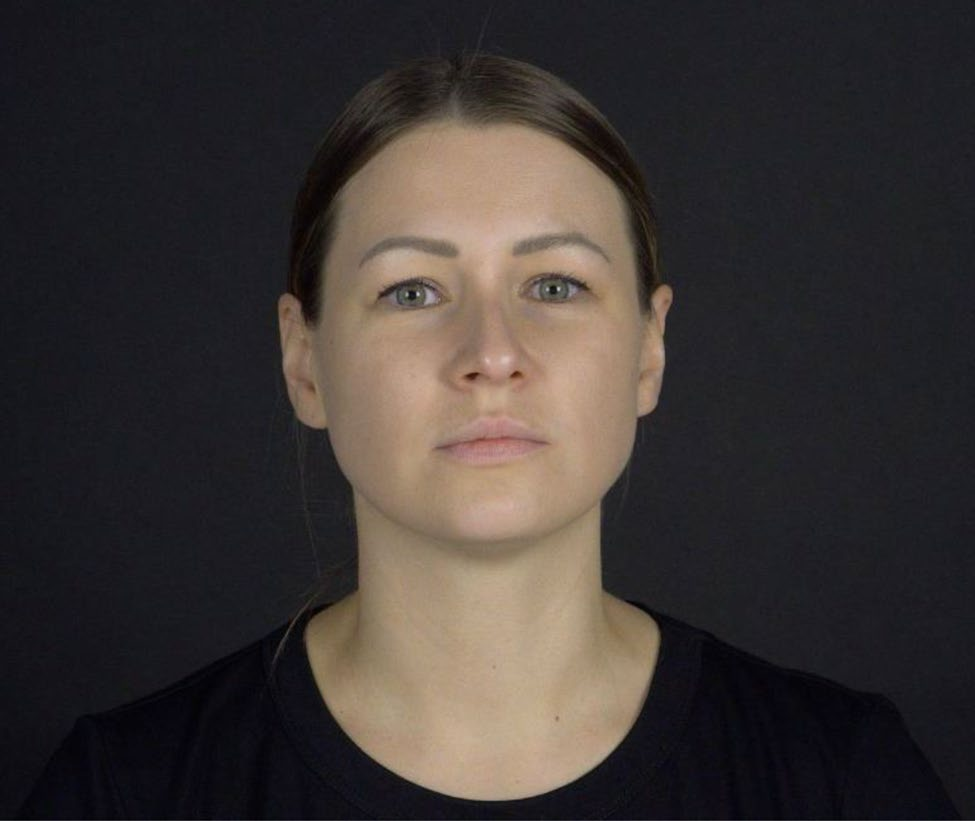
An example frame from the screen-based eye-tracking stimuli. The image is displayed with permission from the actress

The stimuli consist of 9 videos of actresses (1 actress per video) articulating infant-friendly stories and nursery rhymes presented in a fully randomized order (mean duration of each clip = 30.4 s). In all clips, the speaker’s head is centered in the video, providing a full frontal view of the face on a black background (see Fig. 2 for an example). Each video is preceded by a moving picture accompanied by a sound (attention-getter) to focus the infant’s attention on the center of the screen (the approximate location of the actress’s nose). When an infant looks at the attention-getter, the experimenter starts the next trial (max. display time of each attention-getter is 2 s). In total, the entire eye-tracking procedure lasts around 5 min.

#### Screen-based eye-tracking data analysis

The screen-based eye-tracking data will be analyzed using in-house R scripts. The fixations will be extracted using the I2MC toolbox [60]. We will measure the total looking time to three Areas-of-Interest (AOIs): (a) the entire face oval, (b) the eyes, (c) the mouth. The eyes and the mouth AOIs will have a comparable surface area size. Then, the proportion of total looking time (PTLT) for each AOI (mouth vs. eyes) relative to the total looking time to the whole face will be calculated, producing two variables: the ratio of looking time for the eyes compared to the face (PTLT Eyes: Eyes-to-Face Ratio), and similarly for the mouth (PTLT Mouth: Mouth-to-Face Ratio).

### Objective 3. To investigate how the transition to independent sitting associates with infants’ vocal production

Recording of infant-parent interaction from each testing session will be coded offline to annotate infants’ vocalizations using PRAAT software (Boersma and Weenink, 2025, https://www.fon.hum.uva.nl/praat/. The coders will mark the onsets and offsets of each vocalization at the utterance level. An utterance is defined as a vocalization occurring on one expiration cycle [61, 62]. All vocalizations will classified into four distinct, non-overlapping categories (based on [63]): (a) reflexive sounds (laugh and cry), (b) protophones (squeals, vowel-like sounds, growls, whispers, yells, grunts), (c) syllables. The rate per minute, total duration of vocalizing and the mean duration of vocalization will be analyzed.

### Control variables

#### Chronological age and socioeconomic variables

We will use the infant’s chronological age at each testing session as a control variable. Furthermore, we will collect information on the family’s socio-economic status, the infant’s health, the course of pregnancy, and risk factors during the first testing session through a structured interview with the caregiver.

#### Questionnaire data

- **Infant Behavior Questionnaire (Very Short Version)**: Measures infant temperament based on caregiver reports (IBQ-R [64, 65]; Polish adaptation by [66]).
- **Language Use Inventory (only Part A: Gestures)**: Evaluates early language development by assessing a child’s use of gestures as a precursor to verbal communication ( [67]; Polish adaptation by [68]).
- **Early Motor Questionnaire**: Assesses early motor development in infants and young children by evaluating gross motor, fine motor, and action/perception skills based on caregiver reports ( [69]; Polish adaptation by [70]).
- **Parental Beliefs on Motor Development & Motor Habits**: Examines parental attitudes and beliefs regarding children’s motor development and their influence on physical activity and motor habits [71].

Furthermore, given the link between parental mental health and its effects on infant developmental trajectories, with anxiety, depressive symptoms, and stress often having lasting effects on developmental trajectories [72], we decided to also include some control measures of parental mental health. Maternal mental health problems, even in non-clinical samples, have also been shown to influence motor development [73, 74] and are associated with the dynamic organization of infant limb movement [75]. It could potentially relate to how mothers structure their infants’ proximal environment and constrain (or not constrain) their spontaneous movements. They could encourage or discourage the infant’s movement through space (e.g., through the use of devices such as baby bouncers) and provide more or fewer opportunities for their exploration of the environment (e.g [74]).

- **Edinburgh Postnatal Depression Scale**: Screens for symptoms of postnatal depression in parents, focusing on emotional well-being and depressive symptoms [76].
- **State/Trait Anxiety Inventory**: Assesses both temporary (state) and chronic (trait) anxiety levels in parents, distinguishing situational distress from general anxiety tendencies ( [77], Polish adaptation by [78]).
- **Parental Burnout Assessment Questionnaire**: Measures the extent of parental burnout, characterized by exhaustion, emotional distancing, and loss of fulfillment in the parenting role ( [79], Polish adaptation by [80]).

### Measurement of sitting capacity and sitting performance

Following the World Health Organization’s International Classification of Functioning, Disability, and Health (2001) and the approach proposed by Kretch et al. [81], we will differentiate between *sitting capacity* – the potential to perform a task in a standardized or idealized setting – and *sitting performance*, which reflects the actual execution of the task in a natural, everyday environment. Infant’s capacity for sitting will be assessed through the Alberta Infant Motor Scale (AIMS [56]), whereas their *performance* will be evaluated by observing how they sit during dyadic interactions. Thus, the *sitting capacity* represents the ability to carry out a skill under controlled or prompted conditions, while *sitting performance* indicates the unprompted application of that skill in real-world contexts.

### Measurement of *sitting capacity*

To assess infants’ stage of sitting acquisition, we will use the Alberta Infant Motor Scale (AIMS [56]), which is an observational assessment tool for measuring motor development of infants aged 0 to 18 months. It consists of 4 subscales that list skills in four body positions: prone, supine, sit, and stand. For each of the four positions, an experimenter needs to identify and score the least and most advanced items observed during the assessment. The items between the least and most advanced of the observed items represent the infant’s possible motor repertoire in that position, also considered their “window” of current skills. Each item within this window is then scored as either “observed” or “not observed” by the experimenter. All of the “observed” scores on a given subscale are then summed to obtain a positional score. Finally, all positional scores are summed to obtain a total AIMS score. In the planned analyses, we will use both the “sitting” score and the total score to assign infants to one of the three levels regarding sitting experience (pre-sitting, transition, expert).

### Measurement of *sitting performance*

To further confirm infants’ stage of sitting acquisition, we will also analyze the actual time that infants spend in each posture during each parent-child interaction task. Following the previously established procedure [57], we will use the machine-learning classifier (CatBoost) on data recorded with Inertial Motion Units (consisting of accelerometers, magnetometers, and gyroscopes) worn by infants on arms and legs. Inertial Motion Units (MTw Awinda, Xsens Technologies B.V.) connected wirelessly through an Awinda station receiver (Xsens Technologies B.V.) and synchronized in real-time with MT Manager Software (Xsens Technologies B.V.) were recording at 60 Hz. We will specifically focus on the % of the recording time that the infant spent in an independent sitting position, that is, excluding episodes of supported sitting.

### Statistical analyses

Data will be analyzed according to the aims of the study. The use of multiple eye-tracking methods in a single study may increase the risk of data attrition. Hence, we will adopt a flexible policy of participant inclusion on an analysis-by-analysis basis to maximize the sample size for each type of modeling. Thus, linear mixed models will be used to measure the effect of sitting expertise on each outcome variable. These models are suitable for longitudinal designs such as ours and for dealing with potential missing data. Infants’ age will be added to the analyses to control for the interaction between age-related and motor-development-related effects. The linear mixed models take into account the dependency and ordering of the data within subjects in repeated-measures designs. Moreover, even if a subject is missing one or more of the repeated measurements, the remaining data of that subject are used in the analysis.

## Data Availability

Due to the sensitive nature of the video data collected (recording of minors), its sharing will be highly restricted and limited to protect participant confidentiality and privacy. Other types of collected data (i.e., eye-tracking data, data from wearable motion tracking, data from wearable eye-tracker (excluding scene camera recordings that contain faces of caregivers) will be shared in accordance with the European Code of Conduct for Research Integrity and following the principle to be “as open as possible, as closed as necessary.” Additionally, the processing and analysis code developed for the study will be made publicly available on platforms such as OSF and GitHub, promoting transparency and enabling replication of the research while safeguarding the integrity of sensitive data. Finally, the study’s findings will be disseminated widely through scientific publications in peer-reviewed journals.

## Abbreviations

AIMS: Alberta Infant Motor Scale
AOI: Area-of-Interest
ASD: Autism spectrum disorder
EL-infants: Infants at elevated likelihood for autism who have an older sibling with an autism diagnosis
LL-infants: Infants at low likelihood for autism
WHO: World Health Organization
